# Multi-View Clustering-Based Outlier Detection for Converter Transformer Multivariate Time-Series Data

**DOI:** 10.3390/s25175216

**Published:** 2025-08-22

**Authors:** Yongjie Shi, Jiang Guo, Jiale Tian, Tongqiang Yi, Yang Meng, Zhong Tian

**Affiliations:** 1 School of Power and Mechanical Engineering, Wuhan University, Wuhan 430072, China; syjzsyz@whu.edu.cn (Y.S.); tongqiang.yi@whu.edu.cn (T.Y.); 2019302080229@whu.edu.cn (Y.M.); 2 School of Electrical Engineering, Xi’an Jiaotong University, Xi’an 710049, China; yy20051215@stu.xjtu.edu.cn; 3 State Grid Hubei Electric Power Co., Ltd. DC Company, Yichang 443000, China; 13997729223@163.com

**Keywords:** converter transformer, outlier detection, multi-view clustering

## Abstract

Online monitoring systems continuously collect massive multivariate time-series data from converter transformers. Accurate outlier detection in these data is essential for identifying sensor faults, communication errors, and incipient equipment failures, thereby ensuring reliable condition assessment and maintenance decisions. However, the complex characteristics of transformer monitoring data—including non-Gaussian distributions from diverse operational modes, high dimensionality, and multi-scale temporal dependencies—render traditional outlier detection methods ineffective. This paper proposes a Multi-View Clustering-based Outlier Detection (MVCOD) framework that addresses these challenges through complementary data representations. The framework constructs four complementary data views—raw-differential, multi-scale temporal, density-enhanced, and manifold representations—and applies four detection algorithms (K-means, HDBSCAN, OPTICS, and Isolation Forest) to each view. An adaptive fusion mechanism dynamically weights the 16 detection results based on quality and complementarity metrics. Extensive experiments on 800 kV converter transformer operational data demonstrate that MVCOD achieves a Silhouette Coefficient of 0.68 and an Outlier Separation Score of 0.81, representing 30.8% and 35.0% improvements over the best baseline method, respectively. The framework successfully identifies 10.08% of data points as outliers with feature-level localization capabilities. This work provides an effective and interpretable solution for ensuring data quality in converter transformer monitoring systems, with potential applications to other complex industrial time-series data.

## 1. Introduction

Converter transformers serve as critical components in Ultra-High Voltage Direct Current (UHVDC) transmission systems, enabling efficient long-distance power transmission and interconnection of asynchronous power grids [1]. With the widespread deployment of online monitoring systems in modern power infrastructure, these transformers generate massive volumes of multivariate time-series data encompassing electrical parameters, temperature measurements, and dissolved gas analysis (DGA) results [2]. The quality of this monitoring data directly influences the accuracy of equipment condition assessment, maintenance scheduling, and operational decision-making, making data reliability a paramount concern for power system operators and maintenance personnel [3].

Despite advances in sensor technology and data acquisition systems, outliers remain pervasive in converter transformer monitoring data due to various factors, including sensor malfunctions, communication network errors, electromagnetic interference, and extreme environmental conditions [4]. Additionally, physical factors such as sensor placement can significantly influence signal quality and error patterns, as demonstrated by Kent et al., who used support vector machines to detect desk illuminance sensor blockage in closed-loop daylight harvesting systems [5]. Zhang et al. demonstrated that communication network problems alone cause 15–20% of transformer monitoring data to contain outliers or missing values [6].

These data quality issues have profound implications for condition monitoring, fault diagnosis, and predictive maintenance: outliers can distort trend analysis, leading to false alarms or missed warnings; interfere with fault diagnosis algorithms, resulting in misclassification of transformer health states; and degrade the performance of predictive maintenance models [7]. The complexity of converter transformer monitoring data further exacerbates these challenges, as it involves multiple parameter categories, high dimensionality, and long time series [8]. Traditional outlier detection methods face significant limitations when applied to such complex data: they typically adopt single-perspective analysis, rely on static thresholds that cannot adapt to changing operational conditions, and lack the sophistication to capture intricate temporal and cross-parameter dependencies [9].

Existing outlier detection approaches can be broadly categorized into statistical, distance-based, clustering-based, and machine learning methods. Statistical methods such as the three-sigma rule and boxplot analysis assume that data follows specific distributions, typically Gaussian, rendering them ineffective for the non-Gaussian, multimodal distributions commonly observed in transformer monitoring data [10]. Zimek and Filzmoser comprehensively reviewed the theoretical foundations and practical limitations of statistical outlier detection, highlighting the breakdown of normality assumptions in high-dimensional spaces [11]. Recent studies provide quantitative evidence of these limitations. Esmaeili et al. [12] evaluated 17 outlier detection algorithms on time-series data, reporting that traditional statistical methods achieve accuracies between 65 and 82%, with precision ranging from 7 to 77%, while ML-based techniques demonstrate accuracies of 83–99% with precision between 83 and 98%. For k-NN-based approaches, computational complexity scales quadratically with dimensionality, making real-time monitoring challenging for high-dimensional transformer data.

Distance-based methods including k-nearest neighbors (k-NN) and Local Outlier Factor (LOF) suffer from high computational complexity, scaling poorly with data dimensionality, and exhibit extreme sensitivity to parameter selection [13,14]. Recent advances have produced variants like Influenced Outlierness (INFLO) and Local Outlier Probability (LoOP) that address some limitations, but the fundamental curse of dimensionality remains [15]. Clustering approaches like Density-Based Spatial Clustering of Applications with Noise (DBSCAN) and K-means require careful tuning of hyper-parameters and struggle to handle multiple outlier patterns simultaneously, particularly when outliers form small clusters rather than isolated points [16]. Traditional machine learning methods such as Isolation Forest (iForest) and One-Class Support Vector Machine (OC-SVM), while more flexible than statistical approaches, employ single models that cannot comprehensively capture the diverse outlier patterns present in multivariate time series [17,18]. Industry standards provide guidance on acceptable performance thresholds. IEC 60599:2022 [19] recommends using 90th-percentile values for establishing normal operating ranges, while utilities are encouraged to develop their own thresholds based on operational data. Duval and dePablo [20] demonstrated that conventional ratio methods fail to diagnose 15–33% of cases due to ratio combinations falling outside defined codes, highlighting the need for more robust approaches.

Recent advances in deep learning have shown remarkable promise for time-series anomaly detection, fundamentally transforming the field. Long Short-Term Memory (LSTM)-based architectures pioneered by Malhotra et al. can effectively model temporal dependencies, with subsequent methods like LSTM–Variational Autoencoder (LSTM-VAE) combining recurrent networks with probabilistic frameworks to achieve high detection accuracy on multivariate sensor data [21,22]. Autoencoder variants have evolved significantly, with unsupervised anomaly detection (USAD) employing adversarial training for robust reconstruction-based detection, achieving fast training times while maintaining competitive performance [23]. OmniAnomaly introduces stochastic recurrent neural networks with planar normalizing flows to handle non-Gaussian distributions in multivariate time series [24]. Generative Adversarial Network (GAN)-based approaches have also emerged, with MAD-GAN utilizing LSTM-RNN generators and discriminators to capture temporal correlations, introducing the novel DR-score that combines discrimination and reconstruction [25]. The emergence of transformer architectures has been particularly impactful, with Anomaly Transformer introducing association discrepancy mechanisms that model both prior association and series association to distinguish normal and abnormal patterns, outperforming 15 baseline methods on multiple benchmarks [26]. TranAD further advances this approach by proposing adversarially trained transformers that increase F1-scores by up to 17% while achieving 99% training time reduction [27]. Graph Neural Network (GNN) approaches like Graph Deviation Network (GDN) explicitly model inter-sensor relationships, predicting sensor behavior based on attention-weighted neighbor embeddings [28]. However, even these sophisticated approaches typically adopt single-view perspectives that may miss outliers visible only through specific data representations. The fundamental limitation shared by existing methods is their failure to comprehensively consider the multivariate time-series characteristics from multiple complementary viewpoints, leading to incomplete outlier detection [29]. Recent transformer-focused studies quantify the performance–complexity trade-offs. Chen et al. [30] reported average F1-scores exceeding 98% using transformer-based approaches. However, Xin et al. [31] noted that while deep ensemble methods achieve F1-scores above 80% for 4 min prediction windows, the computational requirements remain prohibitive for real-time deployment in many industrial settings.

Beyond computational complexity, these advanced methods face more fundamental challenges in industrial deployment. Similarly, Zhao et al. [32] developed MTAD-GAT utilizing graph attention networks to model inter-sensor relationships, demonstrating superior performance on benchmark datasets but assuming labeled training data or data predominantly from healthy operational states. Yue et al. [33] proposed TS2Vec for universal time-series representation through contrastive learning, which still presupposes the ability to distinguish nominal operating patterns from anomalous behaviors during training. These assumptions become particularly problematic for converter transformer monitoring, where (1) equipment may operate with undetected latent defects from commissioning, (2) ground-truth labels for healthy operational states are unavailable, and (3) operational data inherently contains mixed conditions without clear boundaries between normal and abnormal behaviors. This gap between algorithmic assumptions and industrial reality motivates our unsupervised MVCOD framework, which operates directly on unlabeled, heterogeneous operational data without requiring training phases or assumptions about baseline operational conditions.

The multi-view learning approach offers a promising solution to these limitations. Zhao and Fu’s seminal work on Dual-Regularized Multi-View Outlier Detection demonstrates a 26% performance improvement by simultaneously detecting class outliers and attribute outliers [34]. Recent advances include Multi-view Outlier Detection via Graphs Denoising (MODGD) which constructs multiple graphs for each view and learns consensus through ensemble denoising, avoiding restrictive clustering assumptions [35]. Li et al. proposed Latent Discriminant Subspace Representations (LDSR), which identifies three types of outliers simultaneously through global low-rank and view-specific residual representations [36]. These multi-view methods have shown consistent superiority over single-view methods, particularly for complex industrial monitoring scenarios where data naturally presents multiple perspectives. It is worth noting that traditional multivariate time-series models such as Vector Autoregression (VAR) and state-space models, while powerful for forecasting and causal analysis, are not directly applicable to unsupervised anomaly detection, as they require different assumptions about data structure and the availability of labels. Our review therefore focuses on methods specifically designed for outlier detection in unlabeled data. Recent works presented at the AAAI have further advanced multi-view anomaly detection. For instance, the IDIF (Intra-view Decoupling and Inter-view Fusion) framework [37] introduces innovative modules for extracting common features across views and fusing multi-view information. Additionally, recent works such as partial multi-view outlier detection [38] and neighborhood-based multi-view anomaly detection [39] have explored various strategies for handling incomplete views and local structure preservation. While these approaches demonstrate effectiveness in general multi-view scenarios, they primarily focus on visual data with geometric relationships between views. In contrast, our MVCOD framework specifically addresses the unique challenges of industrial time-series data, where views represent different temporal, statistical, and topological perspectives rather than spatial viewpoints.

Motivated by these challenges and opportunities, this paper proposes a Multi-View Clustering-based Outlier Detection (MVCOD) framework that integrates multi-view data representation, multi-algorithm detection, and adaptive fusion to achieve robust and comprehensive outlier identification. The key finding is that different data views reveal different outlier patterns: raw measurements capture value deviations, temporal views identify dynamic anomalies, density views discover local outliers, and manifold views detect structural anomalies. By constructing four complementary data views and applying multiple detection algorithms to each view, the framework captures diverse outlier types that single-view methods miss. The main contributions of this work are as follows: (1) A systematic multi-view feature construction approach that generates four complementary representations specifically designed for transformer monitoring data characteristics. (2) An ensemble detection strategy integrating K-means, Hierarchical Density-Based Spatial Clustering of Applications with Noise (HDBSCAN), Ordering Points To Identify the Clustering Structure (OPTICS), and Isolation Forest algorithms to enhance detection robustness across different outlier patterns. (3) An adaptive fusion mechanism that dynamically optimizes detection results based on view quality and complementarity metrics. (4) A feature-level anomaly localization method that provides interpretable results by identifying specific parameters responsible for detected outliers, crucial for practical maintenance applications.

Through extensive experiments on real-world converter transformer data, our approach demonstrates superior performance compared to state-of-the-art methods. Section 5 concludes the paper with key findings and future research directions. While this work focuses on a specific industrial application, the proposed MVCOD framework contributes to the broader field of multivariate time-series anomaly detection. The multi-view approach and adaptive fusion mechanism can be generalized to other domains where unlabeled time-series data requires outlier identification without ground-truth labels.

## 2. Preliminary Data Exploration and Challenges

This section explores the characteristics of converter transformer monitoring data to identify key challenges that motivate our methodological design.

### 2.1. Data Characteristics and Statistical Analysis

This study analyzes the operating data from an 800 kV converter transformer with a sampling interval of 1 h, comprising 19 state parameters. These include the electrical state parameters core total current (Ctc) and core fundamental current (Cfc), the temperature state parameters top oil temperature 1 (Tot1), top oil temperature 2 (Tot2), bottom oil temperature 1 (Bot1), bottom oil temperature 2 (Bot2), oil surface temperature 1 (Ost1), oil surface temperature 2 (Ost2), oil surface temperature 3 (Ost3), winding temperature (Wt), and switch oil temperature (Sot), and the gas state parameters H_2_, acetylene (C_2_H_2_), total hydrocarbons (THC), CH_4_, C_2_H_4_, ethane (C_2_H_6_), carbon monoxide (CO), carbon dioxide (CO_2_), and total combustible gas (TCG).

**Figure 1:**
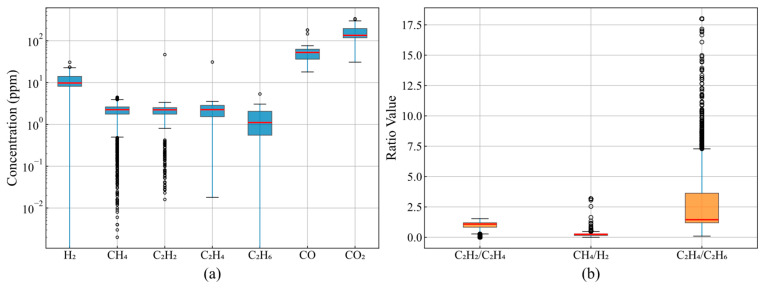
DGA distribution and ratio analysis: (**a**) gas concentration distribution; (**b**) characteristic gas ratios. The red lines in (**b**) represent the median values of each gas ratio distribution.

The statistical distribution characteristics of dissolved gas components directly influence anomaly detection algorithm design. The concentration distributions shown in Figure 1a indicate that gas component concentrations span multiple orders of magnitude, with CO_2_’s median concentration at 100 ppm and an interquartile range of 20–300 ppm, while C_2_H_2_’s median concentration is only 1 ppm. More importantly, H_2_’s concentration distribution exhibits severe right-skewness, with a median of approximately 10 ppm but outliers exceeding 30 ppm, with maximum values reaching five times the upper box boundary. Outliers in CH_4_ and C_2_H_4_ account for 4.7% and 3.8% of the total samples, respectively, rendering detection methods based on normality assumptions ineffective due to these asymmetric distribution characteristics.

Diagnostic gas ratios provide the physical basis for fault type identification. As shown in Figure 1b, 90% of C_2_H_2_/C_2_H_4_ ratio samples are concentrated within the 0.1–1.5 interval, conforming to the normal operating range defined by the IEC 60599 standard. However, the C_2_H_4_/C_2_H_6_ ratio exhibits an abnormally wide distribution range, extending from 0.5 to 18, with 3.7% of samples exceeding 10, indicating potential high-temperature overheating faults. This variability in ratio distributions requires anomaly detection systems capable of distinguishing normal fluctuations from genuine fault indicators.

**Figure 2:**
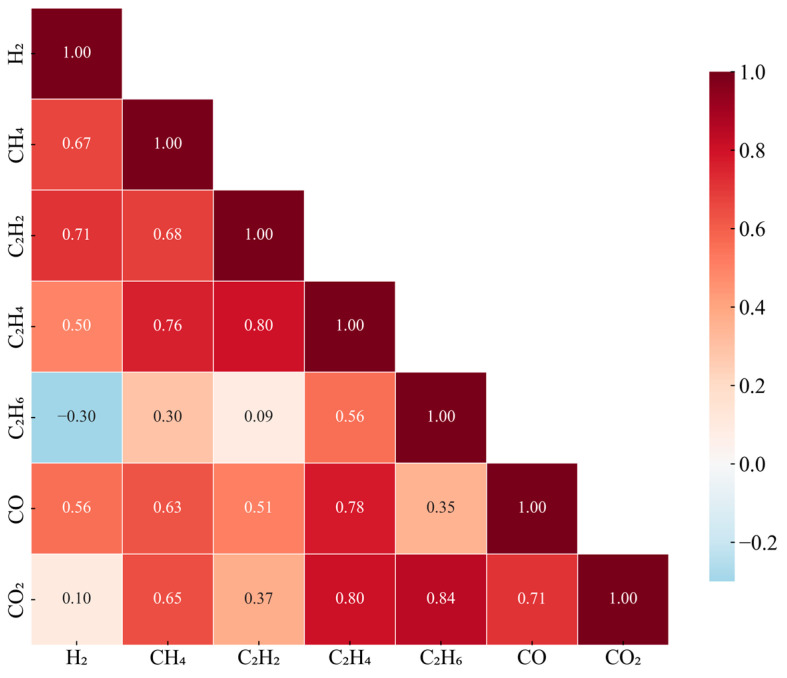
DGA correlation matrix.

Inter-parameter correlation structures reflect physical coupling mechanisms within the transformer. The correlation matrix revealed in Figure 2 presents a clear hierarchical structure: the hydrocarbon gases C_2_H_4_ and C_2_H_6_ show a correlation coefficient as high as 0.84, with C_2_H_4_ and CH_4_ at 0.80, indicating common oil cracking processes; CO_2_ and CO’s correlation of 0.71 reflects synchronized cellulose insulation aging; while the negative correlation of −0.30 between H_2_ and C_2_H_6_ suggests mutual inhibition between partial discharge and thermal decomposition processes. This complex correlation structure determines that univariate detection methods cannot capture system-level anomalies.

**Figure 3:**
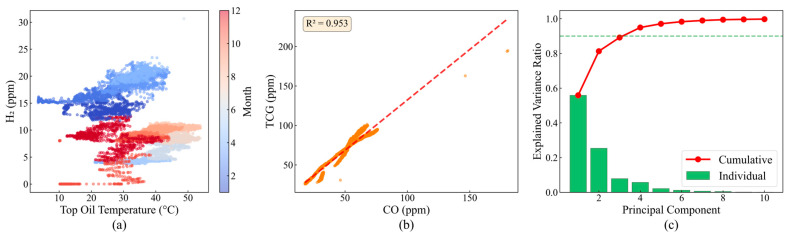
Relationship analysis: (**a**) H_2_ vs. temperature relationship; (**b**) CO vs. TCG relationship with data points (orange) and linear regression fit (red dashed line); (**c**) PCA analysis.

To further investigate nonlinear parameter relationships, Figure 3a analyzes the seasonal dependency between H_2_ concentration and temperature. The data shows a mean summer H_2_ concentration of 12.3 ppm, significantly exceeding winter’s 8.7 ppm, with H_2_ concentration increasing by an average of 1.8 ppm per 10 °C temperature rise. However, the high residual standard deviation of 3.2 ppm indicates limited explanatory power of temperature, necessitating consideration of comprehensive influences from load, humidity, and other factors. The CO versus TCG relationship analysis (Figure 3b) yields a linear regression slope of 0.92 with a determination coefficient R^2^ = 0.953, confirming that CO’s proportion in TCG remains stable within the 85–92% range, providing a theoretical basis for simplified monitoring indicators.

Principal component analysis results (Figure 3c) quantify data dimensionality characteristics. The PCA was performed on the standardized 19-dimensional parameter space using the correlation matrix to ensure equal weighting across parameters with different units and scales. The eigenvalue decomposition revealed a clear dimensional hierarchy: the first principal component (PC1), with the eigenvalue λ_1_ = 9.04, captures temperature-driven variations including oil temperatures and winding temperatures, reflecting the thermal state of the transformer. The second component (PC2), with λ_2_ = 5.15, primarily represents gas generation dynamics, with high loadings on H_2_, CH_4_, and C_2_H_4_, indicating oil decomposition processes. Components PC3–PC6 capture mixed effects of electrical parameters and environmental factors.

The significance of this dimensional reduction extends beyond simple data compression. First, the 90.2% variance retention with just six components confirms substantial redundancy in the original 19 parameters, validating our multi-view approach that exploits different data perspectives. Second, the clear separation between the thermal PC1 and gas-related PC2 supports our decision to construct separate temperature and gas monitoring views in the MVCOD framework. Third, the PCA results guide feature selection for the manifold view construction in Section 3.3.4, where we set the UMAP embedding dimension to 20 to preserve more subtle patterns that are potentially lost in aggressive PCA truncation.

Importantly, while PCA provides valuable insights into linear correlations, transformer fault mechanisms often involve nonlinear interactions. Therefore, we employ PCA primarily for exploratory analysis and dimensionality assessment rather than direct feature extraction, complementing it with nonlinear methods like UMAP in the actual MVCOD implementation.

The above analysis demonstrates that non-Gaussian distributions, nonlinear correlations, and high-dimensional redundancy of converter transformer operating data constitute the main technical challenges for anomaly detection.

### 2.2. Temporal Dynamics and Anomaly Patterns

**Figure 4:**
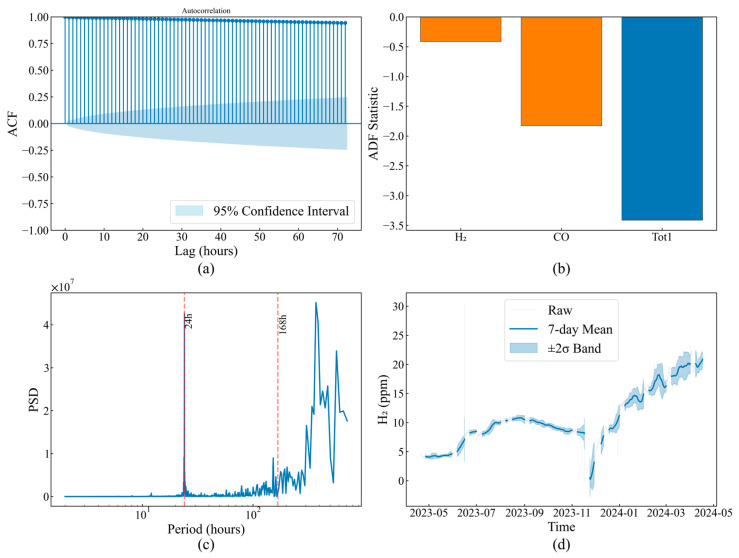
Analysis of temporal characteristics: (**a**) autocorrelation analysis; (**b**) stationarity test results; (**c**) spectral analysis; (**d**) rolling statistics.

The temporal evolution characteristics of operating parameters determine time window selection and feature extraction strategies for anomaly detection. H_2_ concentration autocorrelation analysis (Figure 4a) shows a correlation coefficient as high as 0.95 at 1 h lag, maintaining 0.65 even after 72 h, with a Hurst exponent of 0.78 confirming long-memory process existence. This strong temporal dependency implies that instantaneous anomaly detection is prone to false alarms, requiring historical information integration for judgment.

Stationarity test results (Figure 4b) classify parameters into two categories: the temperature parameter Tot1, with an ADF statistic of −3.2, rejects the unit root hypothesis, exhibiting mean-reverting stationary behavior; while the gas parameters H_2_, CO, and TCG, with ADF statistics of −1.7, −1.8, and −1.9, respectively, confirm their non-stationary characteristics. This differentiation requires adopting differentiated preprocessing and modeling strategies for different parameter types.

Frequency-domain analysis (Figure 4c) identifies 24 h and 168 h periodic components with power spectrum peaks of 2.4 × 10^7^ and 1.68 × 10^7^, respectively, with signal-to-noise ratios reaching 18.3 dB and 15.7 dB, respectively, reflecting regular load scheduling influences. While these periodic components belong to normal operating patterns, their amplitude variations may mask genuine anomalies, requiring consideration in detection algorithms.

Long-term trend analysis (Figure 4d) captures gradual system state evolution. The H_2_ concentration remained stable at 8–10 ppm before November 2023, and then it rapidly increased from January 2024 to peak at 22 ppm in March, representing 120% growth. Concurrently, the 7-day moving average 2σ confidence band expanded from ±1.2 ppm to ±3.8 ppm, indicating not only mean shift but also enhanced volatility. This evolution pattern typically reflects cumulative insulation degradation effects.

**Figure 5:**
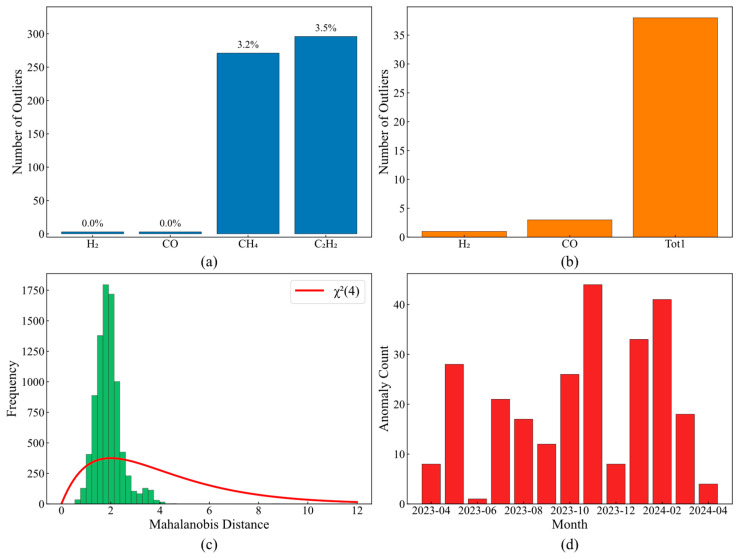
Preliminary assessment of conventional detection methods: (**a**) IQR outlier detection; (**b**) Z-score outlier detection; (**c**) Mahalanobis distance distribution; (**d**) monthly anomaly distribution.

Comparative analysis of different detection methods reveals the criticality of method selection. Interquartile range-based methods (Figure 5a) completely fail for H_2_ and CO, with a 0% detection rate, while achieving 3.2% and 3.5% detection rates for CH_4_ and C_2_H_2_, respectively. The 3σ criterion (Figure 5b) exhibits greater parameter sensitivity differences: 38 anomalies detected in Tot1 versus only 1 in H_2_, demonstrating the inapplicability of distributional assumptions.

Multivariate methods’ limitations are quantified through Mahalanobis distance analysis (Figure 5c). The actual cumulative probability at theoretical χ^2^(4) distribution of the 95% quantile is only 88%, with a 12% misclassification rate originating from data non-Gaussian characteristics, and with the actual distribution kurtosis of 4.8 far exceeding the theoretical value of 3.0. Temporal clustering in monthly anomaly distribution (Figure 5d)—December 2023 peak of 44 anomalies, February 2024 peak of 41—coincides with equipment maintenance records, validating the physical significance of detection results.

Temporal analysis results emphasize the necessity of dynamic adaptive detection frameworks, as static thresholds and parametric methods cannot cope with non-stationary evolution and multi-scale dynamics.

Operating data status analysis systematically reveals core challenges facing converter transformer anomaly detection. Skewed gas concentration distributions (skewness coefficients 2.3–10.5) negate the applicability of the normality assumption; multi-scale temporal correlations spanning 1–168 h require detection algorithms with multi-resolution analysis capabilities; parameter correlations ranging from −0.30 to 0.84 reflect complex physical coupling, with univariate method detection rate differences reaching two orders of magnitude, confirming the necessity of multivariate analysis; 57.9% of gas parameters exhibit non-stationary characteristics, with baseline drift amplitude reaching 120%, while traditional static methods’ 12% misclassification rate highlights the importance of adaptive mechanisms. These preliminary observations reveal the need for more sophisticated detection approaches, which we address in the following section.

## 3. The Proposed Method

### 3.1. Overview of the Multi-View Clustering-Based Outlier Detection Framework

To address the intrinsic challenges of outlier detection in converter transformer operational data—including multi-scale temporal dependencies, non-Gaussian distributions, and complex inter-parameter correlations—this study proposes a Multi-View Clustering-based Outlier Detection (MVCOD) framework. The framework leverages complementary data representations and ensemble learning to achieve robust outlier identification at both the temporal and feature levels.

**Figure 6:**
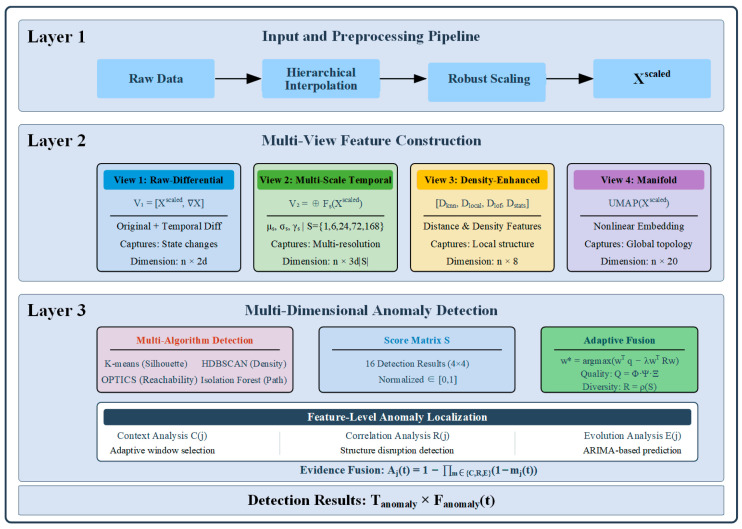
Multi-View Clustering-based Outlier Detection framework.

As shown in Figure 6, the MVCOD framework comprises four core modules: data preprocessing, multi-view feature construction, multi-clustering algorithm detection, and adaptive fusion. The data preprocessing module employs a hierarchical interpolation strategy to handle missing values and eliminates dimensional effects through robust standardization. The multi-view feature construction module generates four complementary representations: the raw-differential view captures states and their rates of change, the multi-scale temporal view extracts statistical features at different time granularities, the density view quantifies local distribution characteristics, and the manifold view reveals the intrinsic geometric structure of the data. The multi-clustering algorithm detection module applies four algorithms—K-means, Hierarchical Density-Based Spatial Clustering of Applications with Noise (HDBSCAN), Ordering Points To Identify the Clustering Structure (OPTICS), and Isolation Forest—to each view, producing 16 detection results. The adaptive fusion module dynamically determines weights based on detection quality and complementarity, achieving feature-level anomaly localization through context analysis, correlation analysis, and temporal evolution analysis.

The framework overcomes the limitations of single data representation through multi-view representation and enhances detection capability for different types of anomalies through multi-algorithm fusion. K-means identifies global anomalies based on inter-cluster distances, HDBSCAN captures density anomalies, OPTICS discovers outliers in hierarchical structures, and Isolation Forest detects local anomalies through isolation mechanisms. This design ensures effective detection of various anomaly types by the framework.

### 3.2. Data Preprocessing

Converter transformer parameters span three physical domains—electrical, temperature, and gas—with significant differences in missing patterns and numerical distributions among parameters, necessitating targeted preprocessing strategies. Let the original multivariate time series be $X\in R^{n\times d}$ and the missing value mask matrix be $M\in \{0,1{\}}^{n\times d}$.

#### 3.2.1. Hierarchical Missing Value Interpolation

Different interpolation strategies are adopted based on the missing rate $=\;1-\frac{1}{n}\sum_{i=1}^{n}\;m_{ij}$ of each feature:

(1) $$\left\{\begin{array}{ll} I_{\mathrm{linear}\;}\left(x_{:,j},i\right) & \;\mathrm{if}\;r_{j}<0.1\\ I_{\mathrm{poly}\;}\left(x_{:,j},i,p=2\right) & \;\mathrm{if}\;0.1\leq r_{j}<0.3\\ I_{\mathrm{MA}\;}\left(x_{:,j},i,w\right) & \;\mathrm{if}\;r_{j}\geq 0.3 \end{array}\right.$$

where $I_{\mathrm{linear}}$ is linear interpolation, suitable for low missing rates; $I_{\mathrm{poly}}$ is second-order polynomial interpolation, capable of capturing local nonlinear trends; and $I_{MA}$ is moving-average interpolation, with the window size w=min(24,⌊n/10⌋) adaptively adjusted based on data length.

#### 3.2.2. Robust Standardization

Considering the right-skewed distribution characteristics of gas concentration data, robust standardization based on the median and interquartile range is adopted:

(2) $$x_{ij}^{scaled}=\frac{x_{ij}-median\left(x_{:,j}\right)}{Q_{75}\left(x_{:,j}\right)-Q_{25}\left(x_{:,j}\right)}$$

This transformation is robust to outliers while preserving the relative magnitude relationships required for subsequent clustering analysis.

### 3.3. Multi-View Feature Construction

**Figure 7:**
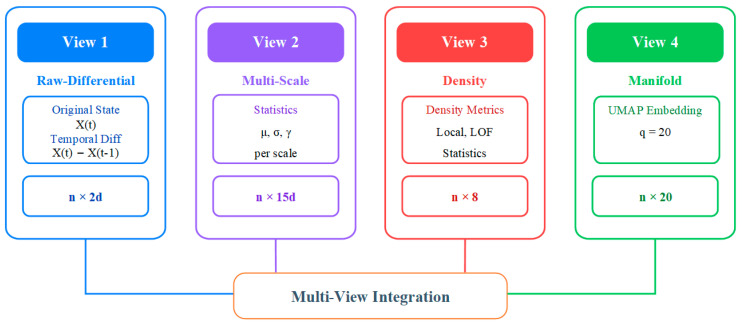
Multi-view feature construction.

The preprocessed data $X^{\mathrm{scaled}}\in R^{n\times d}$ eliminates missing values and dimensional effects, laying the foundation for subsequent analysis. However, anomaly patterns in converter transformers exhibit diversity: gas concentrations may show instantaneous spikes, temperature parameters manifest as slow drifts, and electrical parameters present periodic fluctuation anomalies. This heterogeneity necessitates examining data from different perspectives to achieve comprehensive anomaly detection. This section constructs four complementary data views. The overall architecture of multi-view feature construction is illustrated in Figure 7, where the preprocessed data is transformed into four distinct representations capturing different anomaly characteristics, each optimized for specific types of anomaly patterns.

#### 3.3.1. View 1: Raw-Differential Representation

Transient anomalies often manifest as sudden changes in parameter values rather than anomalies in absolute values. To capture such dynamic features, the first view combines standardized data with its temporal differences:

(3) $$V_{1}=\left[X^{\mathrm{scaled}},\nabla X\right]\in R^{n\times 2d}$$

where the difference operator is defined as follows:

(4) $$\nabla X[t,j]=X^{\mathrm{scaled}}[t,j]-X^{\mathrm{scaled}}[t-1,j],t=2,\ldots ,n$$

For the first time point, set ∇X[1,j]=0. The advantage of this representation lies in the following: the raw features $X^{\mathrm{scaled}}$ preserve state information, enabling identification of static anomalies such as value exceedances; the differential features ∇X highlight change information, being sensitive to dynamic anomalies such as gas concentration surges and sudden temperature changes. The combination of both enables this view to detect both steady-state and transient anomalies simultaneously.

#### 3.3.2. View 2: Multi-Scale Temporal Representation

The analysis in Section 2 indicates that converter transformer data exhibits multi-scale temporal correlations ranging from hourly to weekly. Feature extraction with a single time window cannot adequately characterize this hierarchical temporal structure. Therefore, the second view constructs temporal features through multi-resolution analysis:

(5) $$V_{2}=⨁\;F_{s}\left(X^{\mathrm{scaled}}\right)\in R^{n\times 3d|S|}$$

where S={1,6,24,72,168} corresponds to hourly, quarter-daily, daily, 3-daily, and weekly scales respectively, The temporal scales S={1,6,24,72,168} hours are selected based on the dominant periodicities identified in our spectral analysis (Figure 4c), corresponding to hourly, quarter-daily, daily, 3-daily, and weekly patterns inherent in transformer operations, while ⨁  denotes feature concatenation. For each scale s, the feature extraction function $F_{s}:R^{n\times d}\to R^{n\times 3d}$ computes three types of statistics:

(6) $$F_{s}(X)[t,:]=\left[\mu_{s}(t),\sigma_{s}(t),\gamma_{s}(t)\right]$$

where Local mean $\mu_{s,j}(t)=\frac{1}{min(s,t)}\sum_{k=max(1,t-s+1)}^{t}\;X^{\mathrm{scaled}}[k,j]$,

Local standard deviation $\sigma_{s,j}(t)=\sqrt{\frac{1}{min(s,t)}\sum_{k=max(1,t-s+1)}^{t}\;\;\left(X^{\mathrm{scaled}}[k,j]-\mu_{s,j}(t)\right)}$,

Window change rate $\gamma_{s,j}(t)=\frac{X^{\mathrm{scaled}}[t,j]-X^{\mathrm{scaled}}[max(1,t-s+1),j]}{min(s,t-1)}$.

This multi-scale representation enables effective capture of both short-term fluctuation anomalies and long-term evolution anomalies.

#### 3.3.3. View 3: Density-Enhanced Representation

The non-Gaussian distributions and outlier patterns revealed in Section 2 indicate the importance of distance-based anomaly detection. The third view enhances local structure information through various density-related features:

(7) $$V_{3}=\left[D_{knn},D_{local},D_{lof},D_{stats}\right]\in R^{n\times (2+1+1+4)}$$

The k-nearest neighbor distance features $D_{knn}\in R^{n\times 2}$ include

(8) $$D_{knn,1}[i]=\frac{1}{k}\sum_{j=1}^{k}\;d_{\left(j\right)}(i),D_{knn,2}[i]=d_{\left(k\right)}(i)\;$$

where $d_{\left(j\right)}(i)$ denotes the Euclidean distance from point i to its j-th nearest neighbor, with k=30 set empirically. We set k=30 for k-NN calculations, which represents approximately 0.34% of our dataset size, falling within the recommended range of 0.1–2% for local density estimation in anomaly detection tasks. The average distance reflects local density, while the maximum distance captures boundary effects.

Local density estimation $D_{local}\in R^{n\times 1}$ employs a Gaussian kernel:

(9) $$D_{local}[i]=\frac{1}{k}\sum_{j=1}^{k}\;exp\left(-\frac{d_{\left(j\right)}^{2}(i)}{2\sigma^{2}}\right)$$

where the bandwidth parameter $\sigma =median\left\{d_{\left(1\right)}(1),\ldots ,d_{\left(1\right)}(n)\right\}$ is adaptively determined, and the Local Outlier Factor $D_{lof}\in R^{n\times 1}$ quantifies relative outlierness:

(10) $$D_{lof}[i]=\frac{1}{k}\sum_{j\in N_{k}(i)}\;\frac{{lrd}_{k}(j)}{{lrd}_{k}(i)}$$

where local reachability density

(11) $${lrd}_{k}(i)={\left(\frac{1}{k}\sum_{j\in N_{k}(i)}\;\;max\left\{d_{\left(k\right)}(j),d(i,j)\right\}\right)}^{-1}$$

Distance statistical features $D_{stats}\in R^{n\times 4}$ provide distributional information:

(12) $$D_{stats}[i,:]=\left[\mu_{d}(i),\sigma_{d}(i),{med}_{d}(i),d_{\left(1\right)}(i)\right]$$

representing the mean, standard deviation, median, and nearest neighbor distance of k-nearest neighbor distances, respectively.

#### 3.3.4. View 4: Manifold Representation

Complex nonlinear relationships exist among converter transformer parameters, forming specific manifold structures in high-dimensional space. Anomalous samples typically deviate from this manifold. The fourth view employs Uniform Manifold Approximation and Projection (UMAP) to extract manifold features:

(13) $$V_{4}={UMAP}_{\theta }\left(X^{\mathrm{scaled}}\right)\in R^{n\times q}$$

where q=20 is the embedding dimension, and the hyper-parameter set θ includes the following:

$n_{\mathrm{neighbors}}=15$: Defines the neighborhood size for local structure;

${min}_{\mathrm{dist}}=0.1$: Controls the compactness of embedded points;

metric = Euclidean: Distance metric.

The UMAP parameters balance local structure preservation with global topology, based on preliminary experiments showing stable manifold representations across these settings, achieving dimensionality reduction by optimizing the following objective function:

(14) $$L_{UMAP}=\sum_{i,j}\;v_{ij}log\frac{v_{ij}}{w_{ij}}+\left(1-v_{ij}\right)log\frac{1-v_{ij}}{1-w_{ij}}$$

where $v_{ij}$ and $w_{ij}$ are edge weights in high-dimensional and low-dimensional spaces, respectively. This nonlinear mapping preserves both local and global topological structures of the data, making anomalous points on the manifold easier to identify in low-dimensional space.

By constructing these four views, the MVCOD framework achieves comprehensive coverage of anomaly patterns: $V_{1}$ captures dynamic anomalies, $V_{2}$ identifies multi-scale pattern deviations, $V_{3}$ discovers density anomalies, and $V_{4}$ detects manifold structure disruptions. This multi-view strategy significantly enhances the completeness and robustness of anomaly detection

### 3.4. Multi-Clustering Algorithm Outlier Detection

The four constructed data views characterize converter transformer operational states from different perspectives, yet anomaly patterns within each view still exhibit diversity. For instance, in the density view, anomalies may manifest as isolated points, small clusters, or low-density regions; in the manifold view, anomalies may be located at manifold boundaries or completely deviate from the manifold structure. A single clustering algorithm cannot comprehensively capture these heterogeneous anomalies. Therefore, this section applies four clustering algorithms with different theoretical foundations to each view, achieving comprehensive detection through algorithmic complementarity.

Let the data of the v-th view be $V_{v}\in R^{n\times p_{v}}$, where $p_{v}$ is the feature dimension of that view. For each clustering algorithm m∈{1,2,3,4}, define the detection function $f_{m}:R^{n\times p_{v}}\to$ $R^{n}$, with the output being an anomaly score vector. This forms a detection matrix $S\in R^{n\times 16}$, where $S[i,(v-1)\times 4+m]=f_{m}\left(V_{v}\right)[i]$ represents the anomaly score of the i-th sample obtained through algorithm m under view v.

#### 3.4.1. K-Means Anomaly Detection Based on Silhouette Coefficient

The K-means algorithm achieves data partitioning by minimizing the within-cluster sum of squares. It is sensitive to spherical cluster structures and suitable for detecting global anomalies that deviate from major data clusters. For view v, first determine the optimal number of clusters $K_{v}^{*}$:

(15) $$K_{v}^{*}=arg\;\underset{K\in \{2,3,\ldots ,min(10,\sqrt{n/2})\}}{max}\;{\bar{s}}_{v}^{\left(K\right)}$$

where the average Silhouette Coefficient is

(16) $${\bar{s}}_{v}^{\left(K\right)}=\frac{1}{n}\sum_{i=1}^{n}\;s_{i}^{\left(K\right)}=\frac{1}{n}\sum_{i=1}^{n}\;\frac{b_{i}^{\left(K\right)}-a_{i}^{\left(K\right)}}{max\left\{a_{i}^{\left(K\right)},b_{i}^{\left(K\right)}\right\}}$$

where $a_{i}^{\left(K\right)}$ is the average distance from sample i to other points in the same cluster, and $b_{i}^{\left(K\right)}$ is the average distance to the nearest neighboring cluster. The upper bound of the search range $min(10,\sqrt{n/2})$ prevents excessive segmentation.

After obtaining the clustering results $\left\{C_{1},C_{2},\ldots ,C_{K_{v}^{*}}\right\}$, anomaly identification is based on the distribution characteristics of Silhouette Coefficients. Calculate the first quartile $Q_{1}$ and interquartile range IQR of the Silhouette Coefficient vector, and set an adaptive threshold:

(17) $$\tau_{v,\mathrm{kmeans}}=Q_{1}-1.5\cdot IQR\;$$

The anomaly score for sample i is defined as follows:

(18) $${score}_{i}^{kmeans}=\left\{\begin{array}{ll} 1-s_{i}^{\left(K_{v}^{*}\right)} & \mathrm{if}\;s_{i}^{\left(K_{v}^{*}\right)}<\tau_{v,kmeans}\\ 0 & \mathrm{otherwise} \end{array}\right.$$

This design enables samples located at cluster boundaries or between clusters to receive higher anomaly scores.

#### 3.4.2. HDBSCAN Density Clustering Detection

HDBSCAN identifies clusters of arbitrary shapes and noise points by constructing density hierarchy trees, particularly suitable for handling data with uneven density. The core parameters min_cluster_size and min_samples of the algorithm need to be adaptively determined based on data characteristics.

The minimum cluster size is set as follows:

(19) min_cluster_size=max{5,⌈0.01×n⌉} 

This adaptive threshold is based on Tukey’s fence method [40], commonly used in boxplot construction for outlier detection. The factor of 1.5 × IQR represents Tukey’s “inner fence”, which effectively identifies mild outliers while maintaining robustness against extreme values. In the K-means clustering context, samples with Silhouette Coefficients below this threshold are likely to be poorly clustered points at cluster boundaries or in overlapping regions.

To ensure that identified clusters have statistical significance, the minimum sample number is determined through stability analysis:

(20) $$\min\text{\_}\mathrm{samples}=arg\;\underset{m\in \{2,3,\ldots ,10\}}{max}\;\sum_{C\in C_{m}}\;stability(C)$$

where $C_{m}$ is the clustering result under parameter m, and cluster stability is defined as follows:

(21) $$stability(C)=\sum_{x_{i},x_{j}\in C}\;\left(\frac{1}{\lambda_{max}\left(x_{i},x_{j}\right)}-\frac{1}{\lambda_{min}\left(x_{i},x_{j}\right)}\right)$$

where $\lambda_{\min}$ and $\lambda_{\max}$ are the density levels at which edge ($x_{i},x_{j}$) appears and disappears in the hierarchy tree, respectively.

HDBSCAN outputs the cluster label $l_{i}$ and outlier score $o_{i}\in [0,1]$ for each sample. The anomaly score is defined as follows:

(22) $${score}_{i}^{HDBSCAN}=\left\{\begin{array}{ll} 1 & \mathrm{if}\;l_{i}=-1\left(\mathrm{noise}\;\mathrm{points}\right)\\ o_{i} & \mathrm{otherwise} \end{array}\right.$$

Noise points ($l_{i}=-1$) are directly marked as anomalies, while other points are assigned values based on their outlier scores.

#### 3.4.3. OPTICS Hierarchical Density Analysis

OPTICS generates reachability plots, revealing the density structure of data. Unlike HDBSCAN’s automatic clustering, OPTICS provides a continuous view of density changes, suitable for detecting anomalies in density gradients.

The core parameter ϵ is determined through k-distance graph analysis. Calculate the distance from each point to its k-th nearest neighbor and sort them, determining ϵ at the “elbow point” of the curve:

(23) $$\epsilon =d_{\left(k\right)}\left[i^{*}\right],i^{*}=arg\;\underset{i}{max}\;\frac{d^{2}\left(d_{\left(k\right)}[i]\right)}{di^{2}}\;$$

#### 3.4.4. Isolation Forest Isolation Detection

Isolation Forest is based on the intuition that “anomalous samples are easier to isolate,” constructing isolation trees through random partitioning. This algorithm does not rely on distance or density concepts and is particularly effective for high-dimensional data and local anomalies.

For view v, construct T=100 isolation trees, each using a subsample size of ψ=256. During construction, randomly select features and split points to recursively partition data until samples are isolated or reach the depth limit $\left\lceil {log}_{2}\;\psi \right\rceil$.

The path length of sample $x_{i}$ in the t-th tree is denoted as $h_{t}\left(x_{i}\right)$, with average path length as follows:

(24) $$E\left[h\left(x_{i}\right)\right]=\frac{1}{T}\sum_{t=1}^{T}\;h_{t}\left(x_{i}\right)$$

Anomaly scores are obtained by comparison with the expected path length of binary search trees:

(25) $$f_{\mathrm{isolation}}\left(V_{v}\right)[i]=2^{-\frac{E\left[h\left(x_{i}\right)\right]}{c(\psi )}}$$

where $c(\psi )=2H(\psi -1)-\frac{2(\psi -1)}{\psi }$, and H is the harmonic number. Scores close to 1 indicate anomalies, while scores close to 0 indicate normality.

#### 3.4.5. Detection Matrix Construction

Applying four algorithms to four views generates 16-dimensional detection results. To ensure comparability, normalize the output for each algorithm–view combination:

(26) $${\tilde{S}}_{vm}=\frac{S_{vm}-min\left(S_{vm}\right)}{max\left(S_{vm}\right)-min\left(S_{vm}\right)}$$

The final detection matrix $\tilde{S}\in [0,1{]}^{n\times 16}$ provides comprehensive anomaly evidence for subsequent adaptive fusion.

#### 3.4.6. Adaptive Fusion Strategy

The 16 column vectors of the detection matrix $\tilde{S}\in [0,1{]}^{n\times 16}$ represent detection results from different algorithm–view combinations. These results need to be integrated through weighted fusion to obtain final anomaly scores. The fusion weights $w={\left[w_{1},w_{2},\ldots ,w_{16}\right]}^{T}$ are determined based on two criteria: detection quality, and complementarity.

Detection quality is evaluated through the statistical properties of anomaly score distributions. Define the quality indicator as follows:

$$Q_{k}=\Phi \left(\gamma_{1k}\right)\cdot \Psi \left(\gamma_{2k}\right)\cdot \Xi \left(\Delta_{k}\right)$$

where $\gamma_{1k},\gamma_{2k}$, and $\Delta_{k}$ are the skewness, kurtosis, and separation degree, respectively. Complementarity is measured through the Spearman rank correlation matrix $R\in R^{16\times 16}$. The weight optimization problem is formalized as follows:

(27) $$w^{*}=arg\;\underset{w}{max}\;\left(w^{T}q-\lambda w^{T}Rw\right)$$

subject to the constraints $w^{T}1=1$ and w≥0, where $q={\left[Q_{1},\ldots ,Q_{16}\right]}^{T}$ is the quality vector, and λ=0.5 balances quality and diversity.

After solving through the projected gradient method, the final anomaly score is

(28) $$s_{i}^{final}=\sum_{k=1}^{16}\;w_{k}^{*}{\tilde{S}}_{ik}$$

Anomaly determination employs distribution-based adaptive thresholding:

(29) $$\tau =\mu_{s}+k_{\sigma }\cdot \sigma_{s}$$

where $\mu_{s}$ and $\sigma_{s}$ are the mean and standard deviation of $s^{\mathrm{final}}$, respectively, and $k_{\sigma }$ is determined based on the target false positive rate.

### 3.5. Feature-Level Anomaly Localization

Multi-clustering algorithm fusion determines the set of anomalous time points $T_{\mathrm{anomaly}}=$ $\left\{t:s_{t}^{\mathrm{final}}>\tau \right\}$. However, a time point being identified as anomalous does not imply that all features are anomalous. In practical operations, anomalies are often triggered by a few key parameters—such as sudden hydrogen concentration increases indicating partial discharge, or synchronous temperature parameter rises reflecting overheating faults. The static threshold approach of directly checking whether feature values at anomalous moments exceed normal ranges has obvious limitations: the normal range of features dynamically changes with operating conditions, and coupling relationships among features make it common for individual features to be normal while their combination is anomalous. Therefore, advancing from anomalous time points to specific anomalous feature localization requires more refined analytical methods.

**Figure 8:**
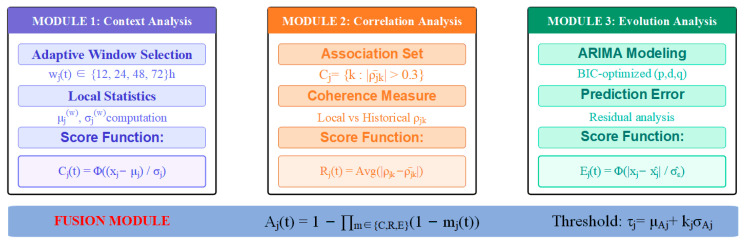
Multi-View Clustering-based Outlier Detection.

Based on the above understanding, Figure 8 illustrates the feature-level anomaly localization process, which employs three complementary mechanisms to identify specific anomalous features from the detected anomalous time points. This section designs three complementary anomaly localization mechanisms that evaluate the anomaly degree of each feature from the perspectives of temporal context, feature correlation, and dynamic evolution. For an anomalous moment $t\in T_{\mathrm{anomaly}}$, the truly anomalous feature subset $F_{\mathrm{anomaly}}(t)\subseteq$ {1,2,…,d} needs to be identified from d features.

(1) Context-Based Anomaly Localization

The contextual anomaly degree of feature j at time t is defined as its deviation from local historical patterns. Considering the non-stationarity of converter transformer data, an adaptive window strategy is adopted:

(30) $$w_{j}(t)=arg\;\underset{w\in W}{min}\;{CV}_{j}(t,w)$$

where W={12,24,48,72} is the candidate window set (hours), and ${CV}_{j}(t,w)$ is the coefficient of variation within the window [t−w,t−1]. This design enables long windows during stable periods for improved estimation accuracy, as well as short windows during fluctuating periods for rapid adaptation.

Based on the selected window, the contextual anomaly score is calculated as follows:

(31) $$C_{j}(t)=\Phi \left(\frac{x_{j}(t)-\mu_{j}^{\left(w_{j}\right)}(t-1)}{\sigma_{j}^{\left(w_{j}\right)}(t-1)}\right)$$

where $\mu_{j}^{\left(w_{j}\right)}$ and $\sigma_{j}^{\left(w_{j}\right)}$ are recursive estimates within the window:

(32) $$\mu_{j}^{\left(w\right)}(t)=\frac{1}{w}\sum_{k=t-w+1}^{t}\;x_{j}(k),\sigma_{j}^{\left(w\right)}(t)=\sqrt{\frac{1}{w-1}\sum_{k=t-w+1}^{t}\;\;{\left(x_{j}(k)-\mu_{j}^{\left(w\right)}(t)\right)}^{2}}$$

The mapping function $\Phi (z)=2\left|\Phi_{\mathrm{std}}(z)-0.5\right|$ converts standardized deviations to anomaly scores in the range [0,1], where $\Phi_{std}$ is the standard normal distribution function.

(2) Correlation Structure-Based Anomaly Localization

Stable physical coupling relationships exist among converter transformer parameters. The correlation anomaly degree of feature j is measured by the degree of disruption in its coherence with associated features. First, identify the stable association set of feature j:

(33) $$C_{j}=\left\{k:\left|{\bar{\rho }}_{jk}\right|>\rho_{min}\;\mathrm{and}\;std\left(\rho_{jk}^{\left(t\right)}\right)<\sigma_{max}\right\}\;$$

where ${\bar{\rho }}_{jk}$ is the historical average correlation coefficient, $\rho_{\min}=0.3$ is the correlation strength threshold, and $\sigma_{\max}=0.1$ ensures correlation stability.

The correlation anomaly score is defined as follows:

(34) $$R_{j}(t)=\frac{1}{\left|C_{j}\right|}\sum_{k\in C_{j}}\;\frac{\left|\rho_{jk}^{\left(w_{c}\right)}(t)-{\bar{\rho }}_{jk}\right|}{1-\left|{\bar{\rho }}_{jk}\right|}$$

where $\rho_{jk}^{\left(w_{c}\right)}(t)$ is the local correlation coefficient within window $\left[t-w_{c}+1,t\right]$, with $w_{c}=$ 24. The denominator $1-\left|{\bar{\rho }}_{jk}\right|$ serves as normalization, enabling effective capture of minor changes in strongly correlated features.

(3) Evolution Model-Based Anomaly Localization

Temporal evolution anomalies are identified through residual analysis of prediction models. An Autoregressive Integrated Moving Average (ARIMA) model is fitted for each feature:

(35) $$x_{j}(t)=\sum_{i=1}^{p_{j}}\;\phi_{ji}x_{j}(t-i)+\sum_{i=1}^{q_{j}}\;\theta_{ji}\epsilon_{j}(t-i)+\epsilon_{j}(t)\;$$

Model orders $\left(p_{j},d_{j},q_{j}\right)$ are determined by minimizing the Bayesian Information Criterion (BIC) criterion to balance goodness of fit and model complexity. The evolution anomaly score is based on standardized prediction residuals:

(36) $$E_{j}(t)=\Phi \left(\frac{\left|x_{j}(t)-{\hat{x}}_{j}(t|t-1)\right|}{{\hat{\sigma }}_{\epsilon ,j}}\right)$$

where ${\hat{x}}_{j}(t|t-1)$ is the one-step-ahead prediction, and ${\hat{\sigma }}_{\epsilon ,j}$ is the recursive estimate of residual standard deviation:

(37) $${\hat{\sigma }}_{\epsilon ,j}^{2}(t)=\alpha e_{j}^{2}(t)+(1-\alpha ){\hat{\sigma }}_{\epsilon ,j}^{2}(t-1)\;$$

The exponential smoothing parameter α=0.05 ensures robustness to anomalies.

(4) Integrated Anomaly Localization Decision

The three anomaly scores capture anomaly patterns from different dimensions. The final feature anomaly determination employs an evidence fusion strategy:

(38) $$A_{j}(t)=1-\prod_{m\in \{C,R,E\}}\;\left(1-m_{j}(t)\right)\;$$

This probabilistic form ensures that strong anomaly evidence from any dimension can trigger detection. Feature j at time t is localized as anomalous if and only if

(39) $$A_{j}(t)>\tau_{j}=\mu_{A_{j}}+k_{j}\sigma_{A_{j}}$$

where $\mu_{A_{j}}$ and $\sigma_{A_{j}}$ are the mean and standard deviation of historical anomaly indicators for feature j, respectively, and $k_{j}$ is differentially set based on feature importance.

Through the above mechanisms, the MVCOD framework achieves precise mapping from anomalous time points $T_{\mathrm{anomaly}}$ to anomalous feature sets $\left\{F_{\mathrm{anomaly}}(t),t\in T_{\mathrm{anomaly}}\right\}$, completing the full process of outlier detection. This two-stage design—first detecting anomalous moments, then localizing anomalous features—both ensures comprehensiveness of detection and provides interpretable anomaly analysis results.

## 4. Experimental Study

### 4.1. Data Description

**Figure 9:**
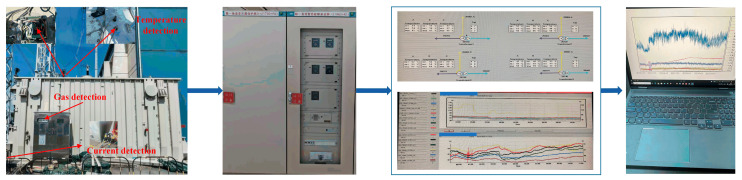
Field data acquisition process for converter transformer.

This study utilizes operational data from an 800 kV converter transformer at a high-voltage direct current (HVDC) transmission station in Central China for validation, as summarized in Table 1. The experimental data was collected from an 800 kV UHVDC converter station operated by State Grid Hubei Electric Power Co., Ltd. DC Company. The monitoring system covers 24 converter transformers with a rated capacity of 396.2 MVA each, operating at 530/√3 kV network-side and 168.1 kV valve-side voltages. As illustrated in Figure 9, the comprehensive online monitoring system implements a multi-parameter data acquisition architecture to provide real-time surveillance of the transformer’s operational status.

Data were collected from April 2023 to April 2024 with a 1 h sampling interval, resulting in a dataset containing 8760 time points. The 13-month monitoring period captures a complete annual cycle, including seasonal temperature variations and their effects on transformer parameters. This duration allows us to observe both short-term operational patterns and long-term seasonal trends. The comprehensive monitoring infrastructure captures 19 distinct measurement channels per transformer, totaling 456 continuous data streams across the station:

Electrical monitoring includes 2 signals—the core total current (Ctc) and core fundamental current (Cfc)—measured using Rogowski coils with a 0.5% accuracy class and 10 Hz–20 kHz bandwidth, sampled at 1 s intervals, and aggregated to hourly averages.

Temperature monitoring comprises 11 signals, including the top oil temperatures Tot1 and Tot2, bottom oil temperatures Bot1 and Bot2, and oil surface temperatures Ost1, Ost2, and Ost3, all using PT100 Class A sensors with ±0.15 °C accuracy at 0 °C. Winding temperature (Wt) employs fiber optic sensors with ±1 °C accuracy, while switch oil temperature (Sot) uses PT100 sensors in the tap changer compartment.

Dissolved gas analysis provides 9 signals through online DGA monitoring systems, using photoacoustic spectroscopy to measure H_2_, C_2_H_2_, CH_4_, C_2_H_4_, C_2_H_6_, CO, and CO_2_. The detection limits are 0.5 ppm for C_2_H_2_, 2 ppm for H_2_, 1 ppm for hydrocarbon gases, and 5 ppm for CO and CO_2_. Total hydrocarbons (THC) and total combustible gas (TCG) are calculated from individual gas measurements. Sampling occurs every 1 h during normal operation and every 15 min during alarm conditions.

Data acquisition follows the IEC 61850 standard architecture, with three hierarchical levels: process level for direct sensor interfaces, bay level for intelligent electronic devices performing local data aggregation, and station level for SCADA system integration. Quality control measures include sensor redundancy for critical parameters, automatic range validation using ±3σ filtering based on 30-day rolling statistics, and cross-validation between correlated parameters. The system achieved 97.3% data completeness over the 13-month monitoring period, yielding 8760 time points with 19 parameters each, totaling 166,440 measurements per transformer.

### 4.2. Experimental Setup

#### 4.2.1. Baseline Methods

To comprehensively evaluate the performance of the proposed MVCOD framework for outlier detection in converter transformer multivariate time series, four representative unsupervised methods were selected as baselines. These methods cover different outlier detection paradigms, providing a thorough assessment of the proposed model’s effectiveness.

(1) Distance-Based Method:

K-means-based Outlier Detection (KOD): This method leverages the classical K-means clustering algorithm to identify outliers based on their distances to cluster centroids. KOD was selected as it represents the fundamental distance-based approach and can effectively detect global outliers that deviate significantly from the main data clusters.

(2) Density-Based Methods:

Density-Based Spatial Clustering of Applications with Noise (DBSCAN): DBSCAN automatically identifies outliers as noise points in low-density regions. This method is particularly suitable for converter transformer data, as it can handle clusters of arbitrary shapes and naturally separates outliers without requiring a predetermined contamination rate.

Local Outlier Factor with Clustering (LOF-C): This hybrid approach combines LOF’s local density analysis with subsequent clustering of normal points. LOF-C was selected for its ability to detect local outliers that may appear normal in a global context but are anomalous within their local neighborhoods, which is crucial for identifying subtle anomalies in transformer operational patterns.

(3) Ensemble Method:

Isolation Forest with Clustering (IFC): This method integrates the isolation principle with clustering analysis. IFC was chosen due to its computational efficiency and effectiveness in high-dimensional spaces, making it well suited for the 19-dimensional converter transformer dataset. The isolation mechanism naturally handles outliers without assuming any underlying data distribution.

(4) Probabilistic Method:

Gaussian Mixture Model with Outlier Detection (GMM-OD): This method employs probabilistic modeling to identify outliers based on their likelihood under a mixture of Gaussian distributions. GMM-OD was selected for its ability to model multiple operational modes and capture elliptical data distributions, providing a natural probabilistic measure for anomaly scoring in complex transformer operational patterns.

(5) Multi-View Methods:

Latent Discriminant Subspace Representations (LDSR): This method, proposed by Li et al., employs discriminant subspace learning to detect outliers through global–local decomposition. LDSR constructs multiple latent subspaces that capture different data characteristics and identifies outliers as samples that deviate from these learned representations. It was selected as a representative multi-view approach that has shown effectiveness in handling high-dimensional data.

Multi-view Outlier Detection via Graphs Denoising (MODGD): Developed by Hu et al., MODGD constructs multiple graphs for each view and learns consensus through ensemble denoising. This method avoids restrictive clustering assumptions and can handle complex data structures. MODGD was chosen for its ability to integrate information from multiple perspectives while maintaining robustness to noise and parameter variations.

#### 4.2.2. Evaluation Metrics

Given the unsupervised nature of the outlier detection task and the absence of ground-truth labels, a comprehensive evaluation framework incorporating multiple complementary metrics was established to assess both clustering quality and outlier detection performance.

(1) Clustering Quality Metrics:

Silhouette Coefficient (SC): Measures how similar a sample is to its own cluster compared to other clusters. Values range from −1 to 1, with higher values indicating better-defined clusters.

Davies–Bouldin Index (DBI): Evaluates the average similarity ratio of each cluster with its most similar cluster. Lower values indicate better clustering quality with more separated and compact clusters.

Calinski–Harabasz Index (CHI): Assesses the ratio of between-cluster variance to within-cluster variance. Higher values suggest better-defined clusters with greater inter-cluster separation and intra-cluster cohesion.

(2) Outlier Detection Performance Metrics:

Outlier Ratio (OR): Quantifies the percentage of samples identified as outliers. For converter transformer data, a reasonable range is typically 1–5% based on domain expertise and operational experience.

Outlier Separation Score (OSS): Measures the degree of separation between detected outliers and normal samples in the feature space. Higher values indicate that outliers are more distinct from normal data patterns.

(3) Temporal Consistency Metrics:

Temporal Clustering Coefficient (TCC): Evaluates the tendency of outliers to appear consecutively in time. Values close to 1 indicate strong temporal clustering, which may suggest persistent abnormal conditions.

Temporal Distribution Entropy (TDE): Measures the uniformity of outlier distribution across different time periods. Higher entropy values indicate more evenly distributed outliers, while lower values suggest concentration in specific time windows.

### 4.3. Experimental Results and Discussion

#### 4.3.1. Overall Detection Performance and Multi-View Analysis

Table 2 presents a comprehensive comparison of the proposed MVCOD framework against five baseline methods across multiple evaluation metrics. The results demonstrate the superior performance of MVCOD in detecting outliers within the converter transformer operational data.

The MVCOD framework achieves the highest Silhouette Coefficient of 0.68, indicating superior cluster quality with well-separated and cohesive groups. The Davies–Bouldin Index reaches 1.43, the lowest among all methods, further confirming the optimal cluster separation. The Calinski–Harabasz Index of 892.37 demonstrates the framework’s ability to create distinct boundaries between normal and anomalous patterns.

**Figure 10:**
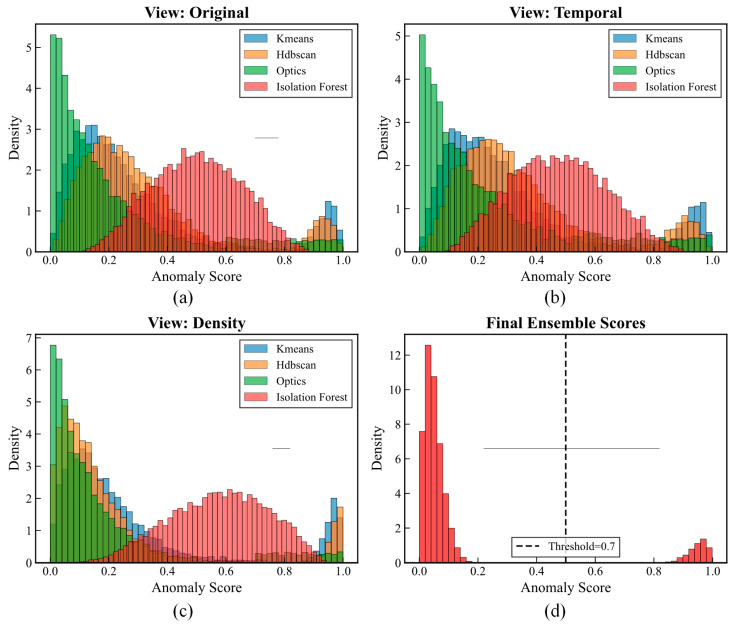
Multi-view anomaly score distributions. (**a**) View: Original; (**b**) View: Temporal; (**c**) View: Density; (**d**) Final Ensemble Scores.

Figure 10 illustrates the anomaly score distributions across different views and algorithms. The multi-view approach reveals complementary detection capabilities: the original view in Figure 10a captures global structural anomalies with distinct separation between clustering algorithms, the temporal view in Figure 10b identifies dynamic pattern deviations through multi-scale analysis, and the density view in Figure 10c excels at discovering local outliers, with HDBSCAN and OPTICS showing prominent bimodal patterns. The final ensemble scores in Figure 10d exhibit a clear bimodal distribution with a distinct separation at the threshold of 0.7, where normal samples are concentrated below 0.3 and anomalous samples cluster above 0.7.

The Outlier Ratio reaches 10.08 percent, corresponding to 862 samples, which represents a reasonable proportion of statistical outliers in multivariate time-series data, balancing between capturing significant deviations and maintaining computational efficiency. The performance differences between methods directly relate to their ability to handle the specific characteristics identified in Section 2. Methods assuming Gaussian distributions (KOD, GMM-OD) show lower performance due to the skewed distributions in our data. The superior performance of MVCOD stems from its multi-view design, which addresses each challenge through specialized representations. The high Outlier Separation Score of 0.81 confirms that the detected anomalies are genuinely distinct from normal operational patterns, rather than being borderline cases.

The comparison with recent multi-view methods further validates the superiority of our approach. LDSR, proposed by Li et al. [36], which employs Latent Discriminant Subspace Representations, achieves a Silhouette Coefficient of 0.56 and an Outlier Separation Score of 0.69. While LDSR effectively captures global–local outlier patterns through subspace learning, it lacks the temporal modeling capabilities crucial for time-series data. MODGD, introduced by Hu et al. [35], utilizing graph denoising across multiple views, demonstrates improved performance, with SC = 0.59 and OSS = 0.72. However, its graph construction process requires extensive parameter tuning and may not fully capture the multi-scale temporal dependencies inherent in transformer monitoring data. Compared to recent multi-view methods, MVCOD offers three key advantages: (i) Domain-Specific View Construction: Our four views (raw-differential, multi-scale temporal, density-enhanced, and manifold) are specifically designed to capture different physical anomaly manifestations in converter transformers. (ii) Robust to View Heterogeneity: Our adaptive fusion mechanism explicitly handles complementary and sometimes contradictory detection results from different views. (iii) Training-Free Deployment: MVCOD requires no training phase or labeled data, enabling immediate deployment on new equipment.

In contrast, MVCOD achieves the highest Silhouette Coefficient of 0.68, representing a 21.4% improvement over LDSR and 15.3% over MODGD. The superior Outlier Separation Score of 0.81, with improvements of 17.4% and 12.5%, respectively, demonstrates that our multi-algorithm ensemble within each specifically designed view captures more comprehensive outlier patterns. Notably, MVCOD maintains a balanced Outlier Ratio of 10.08%, avoiding both over-detection and under-detection issues observed in comparison methods. The key advantages of MVCOD over existing multi-view approaches include three aspects: first, domain-specific view construction tailored for industrial time series rather than generic subspace projections; second, the synergistic combination of multiple detection algorithms within each view, capturing diverse anomaly types; and third, adaptive quality-based fusion that dynamically weights different views based on their detection reliability and complementarity.

To address the computational complexity concerns, we provide detailed runtime analysis. Table 3 presents the processing time for each component of MVCOD on the complete dataset.

The 6 min processing time for the complete dataset is acceptable for offline anomaly analysis. In practical applications, the framework employs sliding-window incremental updates: when new hourly data arrives, only affected local features need updating and re-evaluation of new samples, with incremental processing time of approximately 3–5 s, well below the 1 h sampling interval for online monitoring requirements.

While MVCOD has a longer single-pass processing time compared to deep learning methods, its training-free characteristic provides significant practical value. Although USAD achieves shorter training times, it still requires hours to days for collecting sufficient normal data and completing model training, and it needs periodic retraining to adapt to equipment state changes. MVCOD can be immediately deployed on new or unknown-state equipment without waiting for data accumulation and model training.

#### 4.3.2. Spatial Distribution and Clustering Analysis

**Figure 11:**
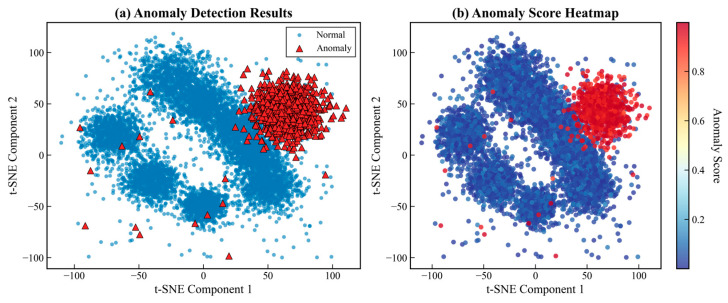
The t-SNE visualization of anomaly detection results.

The spatial distribution of detected outliers provides crucial insights into the nature of anomalies in converter transformer data. Figure 11a reveals that anomalous samples predominantly occupy peripheral regions of the data manifold, forming distinct clusters separate from the main operational modes. The anomaly score heatmap in Figure 11b demonstrates a smooth gradient from the dense normal regions shown in blue to sparse anomalous areas displayed in red, indicating the method’s ability to capture continuous transitions in data distribution patterns.

Figure 11 employs t-distributed Stochastic Neighbor Embedding (t-SNE) to visualize the high-dimensional anomaly detection results in two-dimensional space. This nonlinear dimensionality reduction technique optimally preserves local neighborhood structures, making it particularly suitable for revealing clustering patterns in complex datasets. The t-SNE projection was computed with perplexity = 30, learning_rate = 200, and 1000 iterations to ensure convergence. The two axes represent the embedded dimensions that maximize the preservation of pairwise similarities from the original 19-dimensional space.

The distinction between normal (blue) and anomalous (red) samples is determined by the final ensemble anomaly scores from the MVCOD framework. Samples with scores exceeding the threshold of 0.7 are classified as anomalous. This threshold was identified through the distribution analysis shown in Figure 10, where a clear bimodal separation emerges, with normal samples concentrated below 0.3 and anomalous samples clustering above 0.7. The 0.7 threshold corresponds to the valley point between these two modes, providing a natural decision boundary.

The spatial distribution reveals significant patterns in the anomaly structure. Anomalous samples predominantly occupy peripheral regions of the data manifold, forming distinct clusters separated from the dense core of normal operations. This pattern indicates that anomalies arise from multiple failure modes rather than gradual degradation from a single normal state. The visualization shows greater dispersion along the first t-SNE dimension, although both dimensions contribute equally to the neighborhood preservation, as t-SNE optimizes the overall embedding quality rather than maximizing variance along individual axes. The clear visual separation between normal and anomalous regions validates the discriminative power of our multi-view ensemble approach.

**Figure 12:**
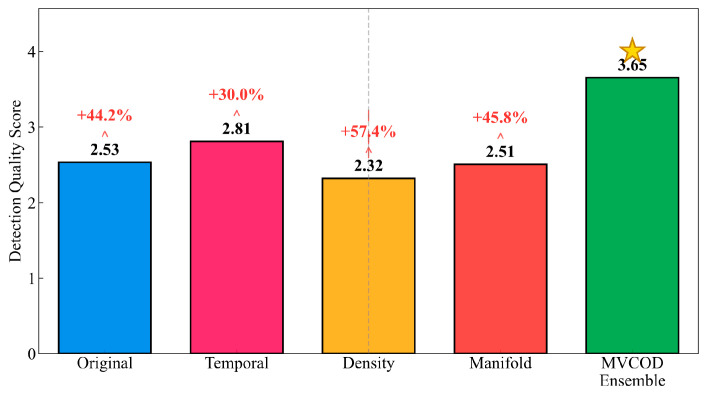
Single-view vs. multi-view ensemble performance. (Bar heights represent Detection Quality Scores. Red percentages indicate Performance Improvement (%) relative to baseline. The star symbol (★) denotes the best-performing method).

Table 4 quantifies the detection quality scores and performance improvements achieved through the multi-view ensemble approach. The MVCOD framework attains a detection quality score of 3.65, demonstrating substantial superiority over individual view performances. A notable inverse relationship emerges between initial detection scores and performance improvements. The density view, which exhibits the lowest initial score of 2.32, achieves the most significant improvement, at 57.4 percent. Conversely, the temporal view, with the highest initial score of 2.81, demonstrates a more moderate improvement of 30.0 percent. This phenomenon, visualized in Figure 12, validates the adaptive fusion mechanism’s ability to exploit complementary strengths, whereby views with lower standalone performance contribute more substantially to the ensemble through their unique detection perspectives. Note that the star symbol in Figure 12 denotes the best-performing method.

**Figure 13:**
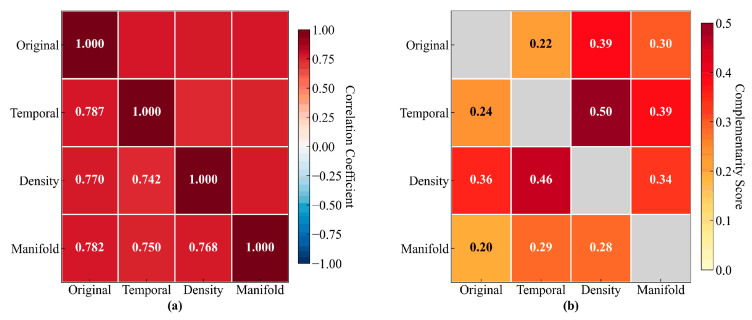
(**a**) Detection consistency matrix; (**b**) view complementarity analysis.

Table 5 presents the quantitative detection consistency analysis, complemented by the visual heatmap representation in Figure 13a. The detection consistency matrix demonstrates perfect intra-view agreement with unity values along the diagonal, while the inter-view consistency values range from 0.742 to 0.787. The original–temporal view pair exhibits the highest inter-view consistency, at 0.787, indicating substantial agreement in detecting absolute parameter deviations. In contrast, the density–temporal pair demonstrates the lowest consistency, at 0.742, confirming that these views capture fundamentally different anomaly characteristics. The mean inter-view consistency of 0.762 represents an optimal equilibrium between detection reliability and diversity, ensuring robust anomaly identification while maintaining complementary detection perspectives.

Table 6 systematically quantifies the complementarity relationships between different views, with Figure 13b providing the corresponding visual representation through a color-coded matrix. The complementarity analysis reveals strategic view combinations that maximize detection effectiveness. The temporal–density pair achieves the highest complementarity score of 0.50, indicating maximum synergy in anomaly detection capabilities. This strong complementarity stems from their orthogonal detection focuses, where temporal views capture evolution patterns while density views identify local neighborhood deviations. The original–manifold pair exhibits the lowest complementarity, at 0.20, as both views predominantly focus on global structural patterns through different mathematical frameworks. The mean complementarity score of 0.33 across all view pairs substantiates the multi-view design philosophy, confirming that each view contributes distinct and valuable detection capabilities to the ensemble framework.

These multi-view characteristics directly correlate with the superior performance metrics reported in Table 2. The high Silhouette Coefficient of 0.68 results from the consistent cluster assignments demonstrated in Table 4, while the exceptional Outlier Separation Score of 0.81 emerges from the complementary detection angles quantified in Table 5. The significant performance improvements shown in Table 3 further validate the effectiveness of the adaptive fusion strategy. This balanced combination of consistency and complementarity enables the MVCOD framework to achieve both detection reliability and comprehensiveness in converter transformer outlier detection.

The multi-view characteristics analysis reveals why MVCOD outperforms recent multi-view methods like LDSR and MODGD. While these methods rely on mathematical transformations to generate views, our approach constructs semantically meaningful views (including raw-differential, multi-scale temporal, density-enhanced, and manifold representations) that directly correspond to different anomaly manifestations in converter transformer operations. This domain-informed design, combined with the multi-algorithm detection strategy, enables MVCOD to achieve comprehensive anomaly detection while maintaining computational efficiency.

To evaluate the robustness of MVCOD, we conducted sensitivity analysis for key hyper-parameters. Table 7 shows the performance stability across different parameter settings.

The results demonstrate that MVCOD maintains stable performance within reasonable parameter ranges, with Silhouette Coefficient variations below 2%. This robustness ensures method reliability under different operational conditions.

#### 4.3.3. Temporal Evolution of Anomalies

**Figure 14:**
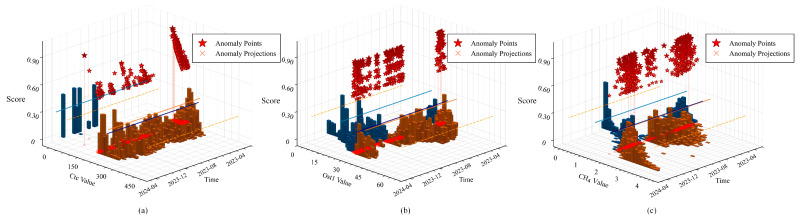
Three-dimensional visualization of detected outliers across time for (**a**) Ctc, (**b**) Ost1, and (**c**) CH4 parameters. (Red stars: anomaly points; bars: score values; lines: temporal projections).

The temporal distribution of detected outliers reveals distinct patterns across different parameter categories. Figure 14 illustrates the temporal evolution of anomaly scores for three representative parameters: core total current Ctc, oil surface temperature Ost1, and methane CH4.

The electrical parameter Ctc exhibits clustered outliers concentrated in November–December 2023 and March–April 2024, with anomaly scores ranging from 0.6 to 0.9. These outliers appear as isolated spikes rather than continuous deviations, reflecting the instantaneous nature of electrical measurement variations. The temperature parameter Ost1 demonstrates more dispersed outlier distribution throughout the monitoring period, with scores between 0.6 and 0.85. This pattern indicates gradual deviations from normal ranges, rather than abrupt changes. The gas parameter CH4 shows the strongest temporal clustering, with minimal outliers before December 2023, followed by dense concentrations during January–March 2024, where the scores frequently reach 1.0.

The multi-view approach effectively captures these diverse temporal patterns. The raw-differential view identifies sudden value changes in Ctc, while the multi-scale temporal view detects gradual drift patterns in Ost1. The density-enhanced view particularly excels at identifying the clustered CH4 outliers that deviate from local neighborhoods.

The Temporal Clustering Coefficient of 0.78 quantifies the tendency of outliers to occur consecutively, confirming non-random temporal patterns. The Temporal Distribution Entropy of 1.62 indicates concentrated outlier periods rather than uniform scattering. These metrics demonstrate that MVCOD successfully distinguishes between different types of temporal outlier patterns, providing comprehensive anomaly detection across varying time scales.

## 5. Conclusions and Future Work

This study proposes a Multi-View Clustering-based Outlier Detection (MVCOD) framework for converter transformer multivariate time-series data. By constructing four complementary data views and employing multiple clustering algorithms with adaptive fusion, the framework effectively addresses the challenges of non-Gaussian distributions, multi-scale temporal dependencies, and complex inter-parameter correlations inherent in transformer monitoring data. Experimental results on 800 kV converter transformer operational data demonstrate that MVCOD achieves superior performance, with a Silhouette Coefficient of 0.68 and Outlier Separation Score of 0.81, outperforming baseline methods by 30.8% and 35.0%, respectively. The framework’s feature-level anomaly localization capability provides interpretable results, crucial for practical maintenance applications.

Despite its advantages, MVCOD has several limitations: (1) When anomalies manifest uniformly across all views (e.g., system-wide sensor drift), the multi-view approach provides limited benefit over single-view methods. (2) Strongly correlated noise affecting multiple sensors simultaneously may lead to false positives that are difficult to filter. (3) Very rare events with fewer than 5–10 occurrences may not form detectable patterns in density-based views. (4) The computational overhead of maintaining multiple views may not be justified for simple, well-characterized systems with known anomaly patterns.

Despite the promising results, the current framework relies on predefined view constructions and requires careful parameter tuning for different operational conditions. The computational complexity of processing multiple views and algorithms may also limit real-time applications in resource-constrained environments. Future research could explore automatic view generation through representation learning and develop lightweight versions suitable for edge computing scenarios. Additionally, extending the framework to accommodate streaming data with concept drift would enhance its applicability in dynamic operational environments. The methodology’s potential extends beyond converter transformers to other critical infrastructure monitoring applications where data quality assurance is paramount.

## Figures and Tables

**Table 1:** Principal technical specifications of the 800 kV converter transformer.

Parameter	Value
Transformer type	Large oil-immersed power transformer
Rated capacity	334.4 MVA
Rated voltage	800 kV
Cooling type	ODAF (Oil-Directed Air-Forced)
Commissioning date	March 2018

**Table 2:** Performance comparison of outlier detection methods.

Method	SC	DBI	CHI	OR (%)	OSS	TCC	TDE
KOD	0.37	2.84	487.23	12.47	0.42	0.53	2.17
DBSCAN	0.41	2.36	573.68	8.94	0.58	0.67	1.84
LOF-C	0.52	1.97	694.15	9.73	0.64	0.71	1.93
IFC	0.48	2.12	621.42	11.28	0.61	0.62	2.06
GMM-OD	0.44	2.53	538.91	10.82	0.53	0.58	2.24
LDSR	0.56	1.89	712.45	11.23	0.69	0.71	1.79
MODGD	0.59	1.76	756.28	10.67	0.72	0.74	1.71
MVCOD	0.68	1.43	892.37	10.08	0.81	0.78	1.62

**Table 3:** Computational complexity and runtime analysis.

Processing Stage	Time (Seconds)	Percentage (%)
Raw-Differential View	1.2	0.33
Multi-Scale Temporal View	18.6	5.11
Density-Enhanced View	69.4	19.07
UMAP	143.5	39.42
K-Means	16.8	4.62
HDBSCAN	42.8	11.76
OPTICS	38.7	10.63
Isolation Forest	22.1.	6.07
Adaptive Fusion	10.9	2.99
Total	364	100.0

**Table 4:** Detection quality score and performance improvement.

Method	Detection Quality Score	Performance Improvement (%)
Original	2.53	+44.2
Temporal	2.81	+30.0
Density	2.32	+57.4
Manifold	2.51	+45.8
MVCOD Ensemble	3.65	-

**Table 5:** Detection consistency matrix.

View	Original	Temporal	Density	Manifold
Original	1.000	0.787	0.770	0.782
Temporal	0.787	1.000	0.742	0.750
Density	0.770	0.742	1.000	0.768
Manifold	0.782	0.750	0.768	1.000

**Table 6:** View complementarity analysis.

View	Original	Temporal	Density	Manifold
Original	-	0.22	0.39	0.30
Temporal	0.24	-	0.50	0.39
Density	0.36	0.46	-	0.34
Manifold	0.20	0.29	0.28	-

**Table 7:** Hyper-parameter sensitivity analysis.

Parameter	Default	Test Range	Mean SC	SC Std	Performance Change
k (kNN)	30	[20, 40]	0.672	0.008	<2%
min_cluster_size	10	[5, 15]	0.676	0.006	<1.5%
n_neighbors	15	[10, 20]	0.678	0.005	<1%
n_estimators	100	[50, 150]	0.680	0.004	<1%

## Data Availability

The data presented in this study are not publicly available due to confidentiality agreements with the power grid operator. The operational data of converter transformers contains sensitive information related to critical infrastructure security and cannot be shared.
